# *Babesia microti*, Upstate New York

**DOI:** 10.3201/eid1103.040599

**Published:** 2005-03

**Authors:** Sarah J. Kogut, Charles D. Thill, Melissa A. Prusinski, Joon-Hak Lee, P. Bryon Backenson, James L. Coleman, Madhu Anand, Dennis J. White

**Affiliations:** * New York State Department of Health, Troy, New York, USA; † New York State Department of Health, Stony Brook, New York, USA; ‡ New York State Department of Health, Albany, New York, USA; § State University of New York at Albany, Rensselaer, New York, USA

**Keywords:** dispatch, babesiosis, Babesia microti, Ixodes scapularis, New York

## Abstract

Five cases of human babesiosis were reported in the Lower Hudson Valley Region of New York State in 2001. An investigation to determine if *Babesia microti* was present in local *Ixodes scapularis* ticks yielded 5 positive pools in 123 pools tested, the first detection of *B. microti* from field-collected *I. scapularis* in upstate New York.

Babesiosis is a malarialike infection often caused in humans by the bite of an infected tick (*1*,*2*). Currently, most cases of human babesiosis in the United States occur in the northeastern and northern Midwest portions of the country and may be attributed to infection with *Babesia microti* (*2*,*3*). *B. microti* is maintained naturally through the same reservoir (the White-footed mouse, *Peromyscus leucopus*) and vector (the Black-legged tick, *Ixodes scapularis*) as *Borrelia burgdorferi*, the etiologic agent of Lyme disease in the United States (*1*,*4*,*5*).

Human cases of babesiosis caused by *B. microti* were first identified in the United States in coastal areas of the Northeast, including several islands off the coast of Cape Cod, Massachusetts; in Rhode Island; and on Long Island, New York (*1*). Studies in Connecticut, Maine, and New Jersey have detected *B. microti* in other northeastern areas. In New Jersey, human cases have been reported from various inland locations across the state (*6*,*7*). In addition, *B. microti* has been identified in local populations of *I. scapularis* from the western portion of the state (*8*). *B. microti* has recently been found in local populations of White-footed mice collected in Connecticut (*9*,*10*). Detection of *B. microti* in Maine has been reported from the Southern Red-backed Vole (*Clethrionomys gapperi*), the Masked Shrew (*Sorex cinereus*), and the Northern Short-tail Shrew (*Blarina brevicauda*) (*11*), as well as from questing *I. scapularis* (*12*).

The first reported case of human babesiosis in New York was from Long Island in 1975 (*13*). Previously, detection of *B. microti* in New York has been limited to small mammals from Shelter Island (off the eastern end of Long Island) (*13*), except for 1 study in 1958 that identified *B. microti* in blood smears taken from a local population of Meadow Voles (*Microtus pennsylvanicus*) in the central portion of the state, near Ithaca (*14*). From 1986, when babesiosis officially became a reportable disease in New York, to 2001, a total of 560 human cases have been reported. Before 2000, most human babesiosis cases were reported from residents of Long Island. Cases reported from residents of upstate New York (north of New York City) are limited; most patients reported travel to locations with a known risk of potential exposure to *B. microti* (*15*). In 2001, 5 confirmed cases of human babesiosis were reported from residents of the Lower Hudson Valley who lived and worked north of recognized risk areas and for whom acquisition of the pathogen by blood transfusion or travel was ruled out (New York State Department of Health, unpub. data). These presumably locally acquired human cases were reported from 4 counties: Columbia (n = 1), Dutchess (n = 2), Putnam (n = 1), and Westchester (n = 1) (Figure).

**Figure:**
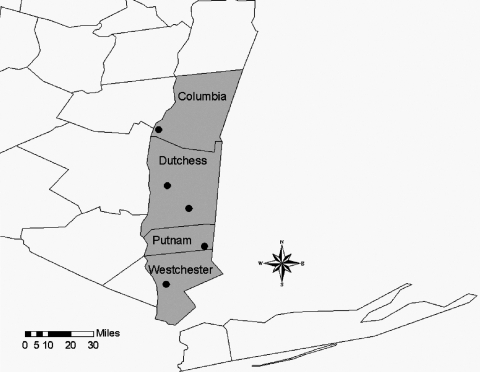
New York State Hudson Valley region. Circles denote generalized locations of tick collection sites in close proximity to locally acquired human babesiosis cases.

## The Study

In response to these presumably locally acquired human cases, an investigation was initiated to detect *B. microti* in local host-seeking populations of *I. scapularis*. Using the limited epidemiologic information available, we chose sites near the residences of suspected locally acquired human case-patients. Typical sites included parks and recreational areas run by state, county, or town governments. All sites were locations in which the possibility of human exposure to potentially infected ticks was considered high. Ticks were collected for 1 hour from each site during spring and fall of 2002 by using a combination of standard techniques, including walking and flagging using a 1-m^2^ piece of white cloth (*16*). All ticks encountered were collected and kept alive until returned to the laboratory, where they were maintained at 4°C until they were sorted by life stage and identified to species (*17*). Specimens were stored in 80% ethanol *I. scapularis* were pooled by location and life stage for testing purposes. Pools consisted of 1 to 10 ticks and were tested for *B. microti* by polymerase chain reaction (PCR) in a blinded fashion. All samples were treated and processed alike, in addition to undergoing the same PCR conditions and analysis.

Briefly, each pool of ticks was homogenized with 125 μL of 5% Chelex-100 resin (BioRad, Richmond, CA, USA) to extract the DNA (the Chelex-100 DNA extraction procedure is the subject of a manuscript in preparation). The primers Bab 1 (5′- CTTAGTATAAGCTTTTATACAGC-3′) and Bab 4 (5′-ATAGGTCAGAAACTTGAATGATACA-3′), targeting the 16S-like small subunit gene (*3*), amplified a product 238 bp in size. Each reaction consisted of 5 μL of 10x PCR buffer (Roche, Indianapolis, IN, USA), 30 pmol of each primer, 1 μL of 2.5 mmol/L deoxynucleoside triphosphate mixture (Roche), 5 U of *Taq* DNA polymerase, and 5 μL of sample. A negative control consisting of 5 μL of nuclease-free H_2_O was included with each run (nuclease-free, reagent quality H_2_O was used throughout to dilute reagents). Known negative tick controls included *Amblyomma americanum*, which do not harbor *B. microti*, and *I. scapularis* from areas where babesiosis is unknown. A positive control consisting of DNA (5 μL) extracted from whole blood of a *B. microti*–infected C3H/HeN mouse (PureGene DNA Blood Isolation Kit, Gentra Systems, Minneapolis, MN, USA) was also included. The PCR was carried out in a GeneAmp PCR System 9700 (Applied Biosystems, Foster City, CA, USA) under the following conditions: 5 min of initial denaturation at 94°C, followed by 35 cycles of denaturation at 94°C (20 s), annealing at 55°C (30 s), and extension at 72°C (30 s). Electrophoresis was carried out on 2% agarose gels, followed by staining with ethidium bromide.

A total of 1,139 *I. scapularis* was collected from 5 locations in the Lower Hudson Valley (Figure). Of the 123 pools tested, evidence of *B. microti* was found in 5 pools of female ticks collected from 3 locations (Table). None of the pools of New York nymphs was positive for *B. microti*. The positive pools collected from Columbia and Westchester Counties each contained 10 females, while the single positive pool from Dutchess County contained 7 female ticks.

**Table:** *Ixodes scapularis* collected in the Lower Hudson Valley Region of New York State and tested for *Babesia microti*

County	Site	Month	Nymphs	Adult	Total	Pools tested	Positive pools
Columbia	A	Jun	52	0	52	6	0
		Oct	1	177	178	19	2
Dutchess	B	Jun	67	2	69	8	0
		Nov	0	72	72	8	0
Dutchess	C	Jun	52	2	54	8	0
		Nov	0	192	192	20	1
Putnam	D	Jun	80	0	80	8	0
		Nov	0	120	120	12	0
Westchester	E	Jul	103	0	103	12	0
		Nov	0	219	219	22	2
Totals			355	784	1,139	123	5

To confirm the identity of each positive PCR product, amplimers were sequenced by using primers Bab 1 and Bab 4. Initial database (GenBank, EMBL, DDBJ) searches for each PCR positive sequence by using MacVector 7.1.1 (Accelrys, San Diego, CA, USA) software (BLASTN, National Institutes of Health, Bethesda, MD, USA) showed high homology with the *B. microti* strain GI 16S-like small subunit rRNA gene. For further confirmation, the sequences were aligned and compared to the *B. microti* 16S-like gene from strain GI reported by Persing et al. (*3*). Homology between the documented 16S-like gene sequence and all 5 PCR products was 100% (not shown). A 60-bp sequence segment, representing all 5 positive specimens, was deposited in GenBank (accession no. AY724679).

## Conclusions

*Borrelia burgdorferi* and *Anaplasma phagocytophila*, the causative agents of Lyme disease and human granulocytic ehrlichiosis, respectively, have been studied more frequently in this region of New York than has *B. microti*. With the discovery of a cluster of human babesiosis cases in the Hudson Valley region, we focused on detecting *B. microti* in vector populations. Finding *B. microti* in local populations of *I. scapularis* provides evidence of locally acquired human babesiosis in the Hudson Valley Region. Since *B. microti* is maintained through the same reservoir and vector species as the causative agent of Lyme disease (*5*), human cases of babesiosis in areas of this state considered endemic for Lyme disease would not be unexpected. The 5 cases represent the first reports of locally acquired babesiosis in residents of New York not living in New York City or on Long Island.

As passive and active surveillance of human disease and tick distribution have demonstrated the continual expansion of Lyme disease and *I. scapularis* throughout New York (*18*), public health authorities should be aware of the potential for an increase in the geographic range of other human diseases transmitted by *I. scapularis*. Accordingly, the New York State Department of Health sent a letter alerting New York physicians to the possibility of patients’ acquiring babesiosis in the lower Hudson Valley. Precautions to prevent tick bites should be adhered to, especially as more information becomes available with regard to the variety of pathogens being transmitted by a single tick species. Further studies to determine the prevalence and distribution of *B. microti–*infected ticks, as well as investigations of simultaneous infection by multiple pathogens such as *B. burgdorferi* and *A. phagocytophila*, are necessary to more readily define the expanding range of *I. scapularis* and the disease agents it harbors.
